# Catalyzing Change: Assessing Inner Setting Context of Cervical Cancer Prevention Efforts in Loreto, Peru, Prior to Transition from VIA to HPV Screen-and-Treat

**DOI:** 10.21203/rs.3.rs-4992569/v1

**Published:** 2024-09-27

**Authors:** Lauren Nussbaum, Joanna Brown, Graciela Meza-Sánchez, Sandra Soto, Magdalena Jurczuk, Javier Vásquez Vásquez, Henrry Daza Grandez, Lita E. Carrillo Jara, Renso López Liñán, Patti E. Gravitt, Valerie A. Paz-Soldán

**Affiliations:** Tulane University School of Public Health and Tropical Medicine; Asociación Benéfica PRISMA; Universidad Nacional de la Amazonía Peruana; Asociación Benéfica PRISMA; Tulane University School of Public Health and Tropical Medicine; Universidad Nacional de la Amazonía Peruana; Gerencia Regional de Salud; Gerencia Regional de Salud; Universidad Nacional de la Amazonía Peruana; National Cancer Institute, National Institutes of Health; Tulane University School of Public Health and Tropical Medicine

**Keywords:** Inner setting, cervical cancer, HPV, Peru

## Abstract

**Background:**

The objective of this study was to understand health care providers’ perspectives regarding the facilitators of and barriers to the success of the former Pap and VIA-based cervical cancer program in Iquitos, Peru, using the Consolidated Framework for Implementation Research (CFIR) to inform the transition to the HPV screen-and-treat intervention. By exploring the pre-implementation organizational context, or inner setting, through the opinions of those who would implement the HPV-based intervention at the patient care level, this research lays the foundation to assess readiness before implementation and understand what’s necessary to design contextually appropriate and sustainable interventions in LMIC settings.

**Methods:**

We conducted 19 semi-structured interviews with health professionals (12 nurse-midwives, 4 doctors, and 3 laboratory technicians) who administered the former Pap- and VIA-based cervical cancer EDT program.

**Results:**

Providers identified information gaps between the primary level of care, where cervical cancer screening occurs, and the hospital level of care, where diagnosis and treatment occurs. These gaps, which were caused in part by fragmented, antiquated, and overlapping data systems, resulted in the loss of patients between levels of care. Participants also noted a lack of trained personnel and basic materials. Some providers found their way around these gaps by facilitating informal information exchanges among providers to ensure women were not lost to follow-up.

**Conclusions:**

PPC relied on these findings and other data from INSPIRE Phase 1 to implement a HPV-based screen-and-treat program that dramatically increased screening and treatment; however, challenges remain regarding resources and sustainability related to HPV technology.

## Background

Cervical cancer is one of the leading causes of female cancer-related mortality in Peru (WHO, 2020), and the Loreto region of Peru experiences a mortality rate 2.6 times higher than the national average (Piñeros et al., 2017). The World Health Organization (WHO) emphasizes the urgent need for the elimination of cervical cancer through implementation of cost-effective interventions, including human papillomavirus (HPV) vaccination and early detection and treatment (EDT) (WHO, 2014). While high-income countries have largely reduced cervical cancer incidence through HPV vaccination and EDT programs, low and middle-income countries (LMICs) like Peru have faced challenges in achieving similar outcomes (Sung et al., 2021; Vale et al., 2021).

Proyecto Precáncer (PPC), an implementation science project aimed to address challenges in cervical cancer prevention by strengthening the EDT services offered in the public health system in Iquitos, Loreto’s capital. Guided by implementation science frameworks, PPC used participatory action research and a systems thinking approach, collaborating closely with stakeholders to implement an HPV-based screen-and-treat strategy with ablative therapy at the primary level (Gilman et al., 2024; Gravitt et al., 2020).

The novel EDT program, launched in July 2019, rapidly transitioned from the previous Pap and visual inspection with acetic acid (VIA) screening to HPV screening with clinician and self-sampling options. The new HPV program shifted the treatment of most HPV-positive women from the hospital level to the primary level of care with the introduction of ablative therapy treatment, with referrals to hospital only for HPV-positive women who were ineligible for ablative therapy. Within six months of implementation, the new HPV-based EDT program surpassed the WHO elimination target of screening 70% of age-eligible women, reaching a peak of 297.7 tests per month (128% of the screening goal) and reducing the loss to follow-up rates from 66.6% (402/604) with VIA to 30.2% (52/172) with HPV screening and treatment (Gravitt et al., 2020; WHO, 2014).

Implementation science, which promotes the uptake of evidence-based practices, plays a crucial role in streamlining the implementation and scale up of new interventions (Bauer et al., 2015), including HPV screening and treatment interventions (Paolino et al., 2023). It aims to reduce the historically long time between discovery of evidence-based interventions and their practical application. By focusing on key factors, including the acceptability, appropriateness, feasibility, and fidelity to the intervention design, implementation research can optimize the future implementation processes so that innovations are effectively and efficiently delivered to those who most need them (Proctor et al., 2011).

Previous studies evaluating implementation of cervical cancer EDT programs in LMICs, including VIA- and HPV-based programs, have identified common barriers to implementation, including: limited awareness of cervical cancer among patients and providers, lack of provider enthusiasm, inadequate resources and infrastructure, high provider turnover, insufficient information exchange, unclear division of labor and responsibilities, disjointed monitoring and evaluations systems, incomplete quality control, antiquated data systems, poor communication, inadequate follow-up and treatment for positive patients, failure to reach rural and mobile populations, lack of national cervical cancer policies, and challenges in maintaining government funding (Atnafu et al., 2024; Holme et al., 2017; Lott et al., 2021; Obol et al., 2020; Paz-Soldán et al., 2012; Robbers et al., 2021; Selmouni et al., 2022; Zhu et al., 2022). Facilitators of implementation include: collaboration between health authorities; political support; guidelines, policies, and training updated to include technological advances; accessible screening services, including counseling and support services; adequate and sustained funding; and leveraging existing sexual and reproductive health program infrastructures (Arrossi et al., 2021; Atnafu et al., 2024; Gilman et al., 2024; Holme et al., 2017; Lott et al., 2021; Robbers et al. 2021; Selmouni et al., 2022; Zhu et al., 2022).

Using the Consolidated Framework for Implementation Research (CFIR) (Damschrolder et al., 2022) to inform the design of and transition to the HPV-based screen-and-treat intervention in Iquitos, the objective of this study was to understand the perspectives of the health care providers regarding the facilitators of and barriers to the success of the former Pap and VIA-based cervical cancer EDT program. The few studies that have used the CFIR framework at the pre-implementation stage have been useful in revealing factors that may impact an intervention’s success (Fokuo et al., 2022; Taylor et al., 2020). By exploring the pre-implementation organizational context, or inner setting, through the opinions of those who would implement the HPV-based intervention at the patient care level, this research lays the foundation for future implementation scientists to assess readiness before implementation and understand the organizational capabilities, tasks, and processes necessary to design contextually appropriate and sustainable interventions in LMIC settings.

## Methods

### Study Setting

Loreto is the largest state of Peru and is located in the northern Amazon River basin. Iquitos (estimated population 442,000 (CPI Research, 2023)), is the largest city in the world that is not accessible by roads; due to the dense surrounding rainforest, it can be reached only by air or boat. Many communities surrounding Iquitos are connected by rivers (Gilman et al., 2024).

This pre-implementation assessment took place in the Micro-Red Iquitos Sur (MRIS) health network, which consists of 17 health facilities with a range services; some with only one nurse-midwife (*obstetra*) and others with multiple doctors, midwives, technicians, and small laboratories. There are two hospitals where women can be referred for tertiary care. The MRIS serves a population of 20,000 women between 30–49 years who are eligible for the screen-and-treat approach using HPV testing and ablative therapy. Most (67%) of the population of Loreto is covered by the *Seguro Integral de Salud* (SIS), a public health insurance program that provides freely available cervical cancer screening and care at the health facilities in the MRIS.

### Participant Selection, Procedures, and Data Collection

During PPC’s time in the MRIS, we gathered data to aid in the implementation of the HPV screen-and-treat program using the INSPIRE model, which has been described in detail elsewhere (Gilman et al., 2024; Gravitt et al., 2020). In short, this model consists of four phases:

- Phase 1: Understanding the Pap and VIA-based cervical cancer EDT program.
- Phase 2: Identifying opportunities to improve the EDT program.
- Phase 3: Strategically implementing a new, internally developed EDT program (the HPV screen-and-treat was the result of this co-design process).
- Phase 4: Continuously monitor and evaluate the new EDT program to learn and adapt.

As part of INSPIRE Phase 1, we conducted this pre-implementation assessment through 19 semi-structured interviews with health professionals. We recruited healthcare workers that were integral to the former Pap- and VIA-based cervical cancer EDT program. All participants worked in the MRIS primary or hospital-level facilities. We interviewed 11 hospital-level and 8 primary-level healthcare staff: 12 midwives, 4 doctors, and 3 laboratory technicians. Our research team contacted participants about the interviews at their work or over the phone, and all participants provided written, informed consent prior to each interview. Between March and October 2017, we conducted 30–60 min interviews with participants in a private area of their facility. Interviews focused on participants’ understanding of the VIA-based EDT program, including their understanding of the flow of information through the system. All interviews were recorded and transcribed.

### Data Analysis

We deductively analyzed participants’ responses using a coding framework based on the updated CFIR domains and subdomains. CFIR, which is used to identify and assess contextual factors that can facilitate or hinder implementation of novel innovations, is made up of five major domains: innovation, outer setting, inner setting, individuals, and implementation (Damschroder et al., 2022). Using Dedoose (Dedoose Version 8.0.35, 2018), two researchers read the 19 transcripts and met to discuss discrepancies in coding and adjust the codebook as needed. One researcher then coded all remaining transcripts, and the research team met to discuss themes that emerged from the data. The research team chose to focus this article on the inner setting domain because that domain and its associated subdomains were the constructs that emerged most frequently from the interviews. Surveys and focus groups with women (individuals domain) and interviews with federal policy makers (outer domain) are reported in other manuscripts (Barrett et al., 2020; Morse et al., 2023; Morse et al., 2024;) or are in progress.

## Results

The inner setting – the focus of this analysis – represents the site-specific organizational culture and structural context for intervention implementation (Fernandez et al., 2018; Fokuo et al., 2022; Walker et al., 2019). The main inner setting subdomains that emerged were work infrastructure, information technology infrastructure, available resources, materials and equipment, communications, access to knowledge and information, and tension for change.

### Work Infrastructure

Regarding how tasks and responsibilities relevant to program implementation are organized among staff members (Damschroder et al., 2022), the first clear barrier was the lack of patient information data flow between providers within the system. Although the referral system is designed for a referral to a higher level of care, and then a counter-referral back to the primary level, that does not occur.

Once she is referred [to the hospital for treatment], I am not informed about what has happened, if she was treated, if she came out positive, or not.... She is now a hospital patient, but I have to follow up with her to see what is happening with her.

—Primary-level midwife

Midwives at the primary level feel responsible for any patient follow-up required, yet they have to either call the hospital midwife for information about their patients or follow up with their patients (and patients are not always able to explain what happened in their follow-up).

Likewise, gynecologists who refer patients to the oncology department of their same hospital for further analysis, or those who refer patients with a cervical cancer diagnosis to Lima for treatment, do not know what happens to their patients.

I don’t know how oncology works, I imagine it works fine, but if I refer a patient to oncology, that’s not great because then we are passive entities. I send the Pap results but I don’t know if the patient will go to oncology…. When I refer her for a biopsy, I don’t perform that biopsy, another doctor does. I don’t know if the patient will go or not, and I lose her.

—Hospital-level doctor

Providers are not formally allocated time and resources to follow up with patients whom they have referred for further screening or treatment at the different system levels. Follow-up is performed at the provider’s discretion; some providers do what they can to fill those gaps so that women are not lost.

This is a tertiary level hospital. We are not a health center. The health centers do the follow-up visits, all of them. We give them a date for their -up visit [at the health centers], 15 days, one month, three months. But if the patient does not attend that appointment, that is her problem. We do not do follow up!

—Hospital-level doctor

Theoretically, everything, VIA and PAP, should be performed at the primary level of care. Here at the tertiary level of care, per Ministry of Health norms, Paps should not be performed here. That’s the ideal, but here we are performing Pap smears so that we don’t lose patients.

—Hospital-level doctor

But patients know that Saturdays are the day for Pap smears. In other words, just Saturdays are for Pap smears and VIA, but if a patient comes for those services during the week, I will do it then, I don’t wait for Saturday…. Perhaps she’s there during the week because that’s when she has time. I try to accommodate the patients that work, because most people here work, whether in their homes, or outside of the city.

—Primary-level midwife

### Information Technology Infrastructure

Regarding the technological systems for data documentation, storage, management, reporting, analysis, and communication support functional performance (Damschroder et al., 2022), the main barrier participants identified in this subdomain was the fragmented health records system. Many primary care health centers used a paper-based system for recording screenings, in part due to a lack of computers or wi-fi at the facilities; some midwives keep their own records in notebooks to keep track of those with whom they must follow up. These physical files were then sent to a single facility, where the files were uploaded to a digital system. However, that digital system often under-recorded the screenings performed, which led to primary care providers being reprimanded for not meeting screening goals, which caused demoralization.

We worked hard last year and the system did not work. This year we are already reviewing the information, we have to show all our work, because it was not fair last year.

—Primary-level midwife

“What are they doing? There are two midwives, and they aren’t doing anything.” In other words, we are always judged, but when we do do the work, nobody calls us to congratulate: “You know what, you’ve done this, congratulations.” Nothing, no recognition.

—Primary-level midwife

For primary care centers that did have digital data systems in place, midwives noted that the systems were difficult to use because they were constantly changing.

They always offer training on how to fill out the [data system], it is always changing. I, for example, have used the [data system] from 2012–2013, I think, then it changed in 2015.

—Primary-level midwife

Further, these IT and paper-based systems were fragmented, overlapping, and not user-friendly.

These documents are the advice and counseling that we give to each patient. And this is our Pap smear registry. Now we have another registry, the VIA registry, with reports and registries for that. In terms of a registry that shows who is due for a Pap this month, who we need to follow up with, we don’t have that specific registry. What we do is we perform follow-up according to the clinical histories. We can see, “last year my colleague did a Pap for this patient, but not for that patient, and here is the result.” On their family planning card, there is a box for Pap smears, if they had one, when was the last time they had one, etc. But a registry specifically for Pap that contains all the patients, and when they are due for the next Pap, we don’t have that.

—Primary-level midwife

### Available Resources

Regarding resources, providers noted a lack of personnel outside of Lima, and specifically in Loreto, who are trained to read laboratory results, provide treatment, or perform needed follow-up care.

I: Do you do conization here too?

P: Of course!

I: That’s what you were trained in last year?

P: Yes, I went to INEN [National Cancer Institute in Lima] for three months.

I: Is anyone else trained in that?

P: No one else.

I: There’s no one else to do conization, for example if you are absent?

P: No one else in this hospital, no one else in Iquitos, there’s no one.

I: And if you are absent?

P: You’re out of luck!

I: So, you’re the only one?

P: Yes, the only one for now, but there will be more! There are more people that can be trained. We are going to send a gynecologist, he was accepted by INEN. He will go in July, for three months.—Hospital-level doctor

I asked a midwife how many Paps the pathologist can read in a day, and she told me 80. Then I asked her how many we send a day, and she said 120–130. So that means, if the pathologist reads 80 per day, 50 remain for the next day, and 100 for the day after that... And maybe that’s the issue, there just isn’t sufficient personnel.—Hospital-level doctor

I: Who is performing Loop Electrosurgical Excision Procedures?

P: Right now, Doctor G. A year and a half ago, we were working with Doctor M. He is a gynecologist with many years of experience, but during that time he was retired. He is someone we were able to bring back, but then, due to a lack of resources, he couldn’t continue. He was working on a contract basis with me, but we haven’t been able to pay him since last September.—Hospital level midwife

I: And when you send them to INEN [for treatment], is there anyone in charge of follow-up for these patients who were referred for treatment?

P: Follow-up, no, we don’t do that, it’s in our work plan to perform follow-up care, but we don’t have the personnel to do that follow-up.

I: You don’t do follow-up.

P: For patients treated in Lima, no. When do we see that patient? When she comes back. And how do we know when she has died? When someone comes to request her death certificate. There is a gaping hole. –Hospital-level midwife

The hierarchical organizational culture is a barrier to creatively addressing the human capacity problem through task-shifting:

I: Who should be trained to perform cryotherapy, only doctors, or midwives too?

P: Don’t be foolish, just doctors! Midwifes do other things. I was in a meeting with midwives last week and it was noted that cryotherapy should be implemented at tertiary and quarternary level facilities. Stick to what you know, a midwife will want to perform up to chemotherapy, but they have their training, in their field. This is the fight they are in! Sometimes I receive prescriptions from midwives here, they should not be prescribing, but they do it anyway. They perform ultrasounds! And the Medical College [of Peru] is very clear that midwives should not be performing ultrasounds. Here I have midwives that are getting their specialty, but they are not allowed to do colposcopies here, and definitely not conization. They have their field of training.

I: But if they were properly trained, could they do it?

P: If the Medical College gives its authorization, they could, but I’m not going to break the rules! That’s not going to happen, at least not in my hospital. I don’t know if they would let that happen in the health centers, it would depend on the doctor there.

I: It’s a question worth asking.

P: You’d have to talk to the Medical College, the midwives want to, but they are not doctors. They say they could do it.... I could perform a cold cone biopsy, but I shouldn’t, that’s what the gynecologists are for! I think I would be capable of performing a cold cone biopsy, I can already do a Loop Electrosurgical Excision Procedure, I think I’m capable of doing a cold cone biopsy, but I won’t, I stick to what I know. — Hospital-level doctor

### Materials and Equipment

In addition to human resources and personnel capacity, participants noted that they lacked materials and equipment that were necessary to implement the EDT program. While doctors noted that they needed equipment to perform treatment, such as biopsy forceps and cryotherapy machines, midwives noted that they often needed various simple supplies, such as examination beds, examination lamps, swabs, vinegar, and specula. The lack of basic supplies to facilitate care was even more apparent at the primary level facilities outside the city center, which can lack consistent running water, electricity, and phone lines. When the health care facilities ran out of their own supplies, it became the patients’ responsibility to provide equipment such as gloves, pain medication, swabs, or even specula.

Sometimes the vagina is wider, longer, and sometimes the speculum cannot reach it, and sometimes we do not have the necessary supplies. They only give us medium size speculums, which might be a bit uncomfortable at the time of performing the pap smear… I have to call the technical staff so that they can give me light and I can reach the cervix.

—Primary-level midwife

They should stock the facility well with biopsy forceps, with everything, because it is not well stocked. We don’t have those things. In other words, if I want to perform a biopsy, I have to stop and tell the patient that I have to coordinate with the laboratory. Sometimes, the lab technician, because he’s a nice guy, will lend me forceps, but that’s something that we don’t have. Thanks to our friendship I can access those things, but we don’t have them.

—Hospital-level doctor

### Communications

Regarding high quality formal and informal information sharing practices within and across the inner setting (Damschroder et al., 2022), we already noted above the information gaps that exist across different levels of care, as well as the incomplete patient records and information systems. However, providers found their way around these gaps by facilitating informal information exchanges among providers through phone calls and text messages.

If I want to refer a patient for a gynecological consult, I pick up the phone and say, “You know what, I have a patient for you.” It’s immediate, so that we don’t lose the patient. Other providers call me when they have someone for me to see, I tell them, “Sure, have her come right now.” Because if we depend on the administrative side, going through the referral process, the patient will first have to make an appointment.

—Hospital-level doctor

To facilitate things a bit, we’ve created a WhatsApp group, so that my colleagues already know who is in charge [of cervical cancer] and that we work here, so when they have a referral, they call me and say, “I’m sending you a patient!” Sometimes the patient comes straight here with her referral.

—Hospital-level midwife

### Access to Knowledge and Information

Concerning the extent to which guidance and training are accessible to system actors to implement and deliver the innovation (Damschroder et al., 2022), overwhelmingly, providers noted that they needed more frequent trainings that were more consistent in terms of content, as well as a system for quality control.

The trainings should be more frequent, because it doesn’t always stay in your head, and things are constantly changing. For example, regarding the light bulb, I didn’t know white light was better for VIA, and so after that training, I changed my light bulb from a yellow light to a white light, but then they told me no, that I should continue with the yellow light bulb. I responded, “But in a training in 2012 you told us to use white light,” but then in 2016, it was yellow light. But I wasn’t able to attend that later training, so I didn’t know that.... The trainings should be annual.

—Primary-level midwife

INEN came to train a group in 2013. I was part of that first group to be trained. Then they formed trainers. Here in the region there are five trainers for VIA, Pap, cryotherapy. They are the ones who are in charge of training all of us, and now there are many midwives who are trained. In San Juan there are many who are trained in VIA and cryotherapy, and they will keep scaling up that training. The goal is that we all speak the same language.

—Hospital-level midwife

For me, the best thing that we could do is strengthen the basic skills regarding taking samples. Supervising, that would take three or four months. And then, after ensuring that everyone is performing well, to have an executive director that monitors the quality. You have to perform quality control using the reports that the system provides. The [2001] Bethesda System is a very good system that allows you to supervise the samples being taken.

—Laboratory technician

### Tension for Change

This subdomain refers to references or perceptions about current circumstances that are intolerable and need to change (Damschroder et al., 2022). Here, providers recognized that the fragmentation between screening and treatment created bottlenecks and gaps in timely treatment that then led to patients lost. Some hypothesized that a superior data system would facilitate improvement.

If we had [an adequate] registry, we would see where things get stuck: where there are delays, if there has been a delay in the referral, in the treatment. Well-managed statistics, or records in this case, would allow us to see the reality of the work we are doing.

—Hospital-level doctor

Others acknowledge the lack of personnel trained to read samples was causing delays, and suggested investing in this human resource and decentralizing it.

Here, there’s a large number of samples to be read, but only one person can read them… I am convinced – and I think we all have the same opinion – that by training more personnel and gathering them to set up the laboratory here, this whole problem would be solved. One, because we would no longer be sending samples to the Regional Hospital… we would do it here.

—Hospital-level doctor

Similarly, others suggested shifting treatment to the primary level of care would improve the program, but identified the policy barriers associated with that change.

The primary level has to be well reinforced, because the primary level does not solve anything…. In the primary level there should be, for example, colposcopies, that’s important, a well-trained doctor, VIA for everyone, Pap for everyone, but that has to be a policy of the [regional health system], of the Ministry!

—Hospital-level doctor

Here there should be at least three or four clinical oncologists and even a surgical oncologist, that’s what we should have. We have asked the Ministry of Health, asked [traveling oncologists] to come one week a month to resolve those cases, to operate from here, for example, a tumor or a cervical cancer that they can operate on here. We have asked the Ministry for this, we gave up for awhile, but we are trying again.

There should be a clinical oncologist here because we are sending people to Lima, but the referral process is terrible, it’s through private clinics.

—Hospital-level doctor

I: When the patient comes and you treat her, how much time passes until the patient has to return to the health post to do her follow-up? Do you do a counter-referral?

P: We don’t do that, but we should! But we don’t! We have a counter-referral sheet for the health posts, but we just don’t have enough personnel to do the counter-referrals.

I: For example, if a patient has cervicitis or vaginal discharge, you treat her, right? Does her health post know that this patient is receiving treatment for cervicitis, and not for a pre-cancerous problem?

P: The health post doesn’t know, that’s what I’m saying, we do not do counter-referrals! If she goes back to her health post, that health post is not going to know firsthand that it is not cancer or a dysplasia.

I: Who is in charge of giving the results, is that you?

P: Pathology gives us the biopsy result.

I: And how does that result get to the health post?

P: It doesn’t, because those results are from here. Everything stays here. But I make a copy of the result and I give it to the patient so that she can have it, I give it to her with her reference sheet from the visit so that she can take it, but we don’t send a counter-referral. We should send that with the patient as well, but we don’t do that because it would take time and money. Someone would have to fill out the counter-referral sheet and we don’t have personnel for that, we are just a few people here!—Hospital-level doctor

## Discussion

This study used CFIR’s inner setting domain to identify the organizational level barriers associated with the former Pap and VIA-based cervical cancer screening and treatment program in Loreto, as well as facilitators to implement an HPV-based screen-and-treat program that would shift treatment to the primary level of care. Many of the challenges associated with the Pap and VIA program were a result of the fragmentation between screening, performed at the primary level, and treatment, performed at the hospital level. Patients were lost to follow-up due to a lack of open communication and data-sharing between the midwifes screening patients and the hospital doctors treating them, as well as the length of time between screening and treatment (Paz-Soldán et al., 2012; Morse et al., 2024). This problem is not unique to Peru and has consistently been highlighted in the literature regarding implementation of screen-and-treat programs in resource-limited settings (Holme et al., 2017; Lott et al., 2021; Selmouni et al., 2022; Thomson et al., 2020). Shifting the treatment responsibility to the primary level of care through ablative therapy would allow for more efficient treatment and conserve hospital-level resources for the patients who require a more advanced level of care (Holme et al., 2017; Poli et al., 2020). Indeed, PPC data have shown that post-intervention screening rates surpassed WHO’s 70% monthly coverage targets within 6 months, as compared to the pre-implementation state, when only 31% of women were being routinely screened with either Pap or VIA; regarding treatment rates, PPC data have shown that a completion of care endpoint was reached by 67.4% of HPV-positive women within 6 months, compared with 30.2% of VIA-positive women at hospital facilities (Gilman et al., 2024).

Further, participants expressed significant frustration with the health records and data system, which impeded their inability to track their patients as they moved across different levels of care. A data system that is consistently accessible to all involved providers and provides comprehensive and accurate data would provide needed data transparency so that health care providers can identify patients who need treatment and/or follow-up care but have not yet received it, including follow-up screenings (Holme et al., 2017; Morse et al., 2024; Thomson et al., 2020; Zhu et al., 2022). PPC addressed this barrier in the intervention by facilitating the design of a triple-copy form that both satisfied health system documentation needs and facilitated adequate patient tracking between levels of care (Gilman et al., 2024).

Organizational culture, specifically a hierarchical structure where norms and practices are mandated from above, without input from those on the ground, is another barrier to successful implementation (Simwinga et al., 2024). However, many evaluations regarding the implementation of cervical cancer prevention programs in LMICs are based on the opinions of policy-makers and other high level stakeholders (Arrossi et al., 2021; Obol et al., 2020; Selmouni et al., 2022), or patients (Hussein et al., 2024; Poli et al., 2020). The few investigations we found that focused solely on provider perspectives were in Africa (Lott et al., 2021; Moucheraud et al., 2020) and Asia (Zhu et al., 2022), not Latin America.

Because implementation is more successful and sustainable when all key stakeholders are involved (Fokuo et al., 2022; Lopze et al., 2024), PPC tackled the hierarchical culture by continuously engaging stakeholders at all levels of the system to ensure wide buy-in (Gilman et al., 2024). Importantly, as described in other studies as well (Arrossi et al., 2021; Obol et al., 2020; Selmouni et al., 2022), most policies are developed at the national Ministry of Health – in the nation’s capital, a very different context, with limited attention or detail about potential site-specific adaptation that is needed for comprehensive implementation and/or scale-up.

Participants emphasized that existing resources—regarding personnel as well as equipment—were insufficient to support implementation of the EDT program. Shifting from a pap and VIA-based screening program to an HPV-based program does not necessarily solve these problems; rather, both methods have their advantages and disadvantages (Holme et al., 2017; Lott et al., 2021). Specifically, an HPV-based program relieves some burden on the human capacity needed to analyze samples by shifting that work to a machine, and substitutes a subjective test with an objective one, which addresses VIA’s issues regarding quality control (Holmet et al., 2017; Moucheraud et al., 2020; Poli et al., 2020; Selmouni et al., 2022). A VIA-based program is attractive due to its low-costs because it does not require advanced technology (Lott et al., 2021; Paz-Soldán et al., 2012), while an HPV-based program requires an expensive machine to read the DNA samples, as well as specific cartridges and cervical swabs that have been difficult for PPC to consistently procure. Therefore, challenges remain regarding access to needed materials for an HPV-based program. This reality reinforces the conclusion that the impact of introducing improved technologies in low-resource settings may be limited if the intervention is discrete, that is, not accompanied by broader efforts to strengthen the health system as a whole (Moucheraud et al, 2020).

Some participants explained how, despite all these challenges, they followed through for their patients by creating informal information channels among providers, tracking down patients to ensure they weren’t lost between the different levels of care, even chipping in for transportation so patients could travel to Lima for treatment. An enthusiastic, committed work force is crucial to motivating women to get screened in the first place; finding leverage within the existing system to opportunistically identify patients who are already seeking health care is of utmost importance in a resource-limited setting (Selmouni et al., 2022). These health care providers are the health care system’s champions that are crucial to PPC’s success specifically, as well as to the adoption and sustainment of new cervical cancer screening and treatment programs in general (Gilman et al., 2024).

This study had several limitations that should be considered. First, by using purposive sampling and focusing only on the inner setting domain through the perspectives of providers, our findings provide only a partial perspective of the barriers to and facilitators of an HPV-based screening and treatment system and may not be representative (Moucheraud et al, 2020). Second, our sample size of health care providers was small, limiting the generalizability of our findings. However, these data were validated by these participants and other stakeholders through the system audits, process mapping, and group model building workshops conducted during INSPIRE Phases 1 and 2 (Gilman et al., 2024; Morse et al., 2024).

## Conclusion

This study used CFIR’s inner setting sub-domains to understand health care providers’ perspectives regarding the facilitators of and barriers to the success of the former Pap and VIA-based cervical cancer EDT program to inform the design of and transition to the HPV screen-and-treat intervention in Iquitos, Peru. Providers identified information gaps between the primary level of care, where cervical cancer screening occurs, and the hospital level of care, where diagnosis and treatment occurs. These gaps, which were caused in part by fragmented, antiquated, and overlapping data systems, resulted in the loss of patients between levels of care. Participants also noted a lack of trained personnel, as well as a lack of basic materials and supply. Some providers found their way around these gaps by taking the initiative to facilitate informal information exchanges among providers to ensure women were not lost to follow-up. PPC relied on these findings and other data from INSPIRE Phase 1 to implement a new HPV-based screen-and-treat program that moved the needle on screening and treating more women; however, challenges remain regarding resources and sustainability related to HPV technology. This research lays the foundation for future implementation science researchers to assess inner setting readiness before implementation to understand the organizational capabilities, tasks, and processes necessary to design contextually appropriate and sustainable interventions in LMIC settings.

## Data Availability

The dataset supporting the conclusions of this article are available upon reasonable request and with permission from the participants.
